# Experimental data for aluminum removal from aqueous solution by raw and iron-modified granular activated carbon

**DOI:** 10.1016/j.dib.2018.01.063

**Published:** 2018-01-31

**Authors:** Mokhtar Mahdavi, Afshin Ebrahimi, Amir Hossein Mahvi, Ali Fatehizadeh, Farham Karakani, Hossein Azarpira

**Affiliations:** a Environmental Health Engineering, Saveh University of Medical Sciences, Saveh, Iran; b Social Determinants of Health Research Center, Saveh University of Medical Sciences, Saveh, Iran; c Department of Environmental Health Engineering, School of Health, Isfahan University of Medical Sciences, Isfahan, Iran; d Environment Research Center, Research Institute for Primordial Prevention of Non-communicable Disease, Isfahan University of Medical Sciences, Isfahan, Iran; e School of Public Health, Tehran University of Medical Sciences, Tehran, Iran; f Center for Solid Waste Research, Institute for Environmental Research, Tehran University of Medical Sciences, Tehran, Iran; g Manager of Passive Defense of Water & Wastewater Engineering Company, Tehran, Iran

**Keywords:** Aluminium removal, Adsorption, Iron-modified GAC, Water treatment

## Abstract

This dataset deals with the modification of granular activated carbon (GAC) with FeCl_3_ under basic conditions (pH ≈ 12) for removal of aluminium (Al) from aqueous solution. The structural properties and operational parameters including Al ion concentration (2.15 and 10.3 mg/L), pH solution (2–10), adsorbent dosage (0.1–5 g/L), and contact time (0–10 h) was investigated for raw and modified GAC. This dataset provides information about Al removal by GAC and modified GAC at conditions including: pH = 8, contact time = 6 h, initial Al concentration = 2.15 mg/L. The characterization data of the adsorbents was analysed by Fourier transform infrared (FTIR) spectroscopy, scanning electron microscopy (SEM) and Brunauer, Emmett and Teller (BET) test. The data showed that Freundlich isotherm with and Pseudo second order kinetic model were the best models for describing the Al adsorption reactions. The acquired data indicated that the maximum adsorption capacity of GAC and modified GAC to uptake Al (*C*_0_ = 10.3 mg/L) was 3 and 4.37 mg/g respectively.

**Specifications Table**

**Table:** 

Subject area	Environmental Engineering
More specific subject area	Adsorption
Type of data	Table, image and figure
How data was acquired	– GAC was oxidized by nitric acid and concentrated sulphuric acid. Then it was modified by FeCl_3_. 6H_2_O under basic condition according to a designed procedure. – Experiments were conducted according to a designed procedure of analytical test and were investigated in order to perform an analysis of adsorption process. All adsorption tests were done in batch mode. – Fourier transform infrared (FTIR) spectroscopy (Shimadzu 4300), scanning electron microscopy (SEM, Hitachi, SU 70) and Brunauer, Emmett and Teller (BET) tests were used to determine the characteristics of the adsorbent. – The aluminium concentration was measured by DR5000 Spectrophotometer (Method 8012) that was adapted from Standard Methods for the Examination of Water and Wastewater.
Data format	Raw and analysed
Experimental factors	Studying variables including pH, contact time, Al concentration, adsorbent dosage and characterisation of raw and modified GAC which were investigated for Al removal by adsorption.
Experimental features	– Characterization data of raw and modified GAC obtained from FTIR, BET and SEM are given. – Optimization of Al adsorption onto raw and modified GAC adsorbent by modification.
Data source location	Saveh University of Medical Sciences.
Data accessibility	The data presented in this article is not published anywhere else.

**Value of the data**

• The data are beneficial for determination of the isotherm and kinetic for predicting and modelling the adsorption capacity and mechanism of Al removal by the iron-modified GAC.
• These data show the efficacy of modified GAC in comparison to raw GAC on Al removal.
• The dataset will be useful for Al removal from aqueous solution.

## 1 Data

**Fig. 1:**
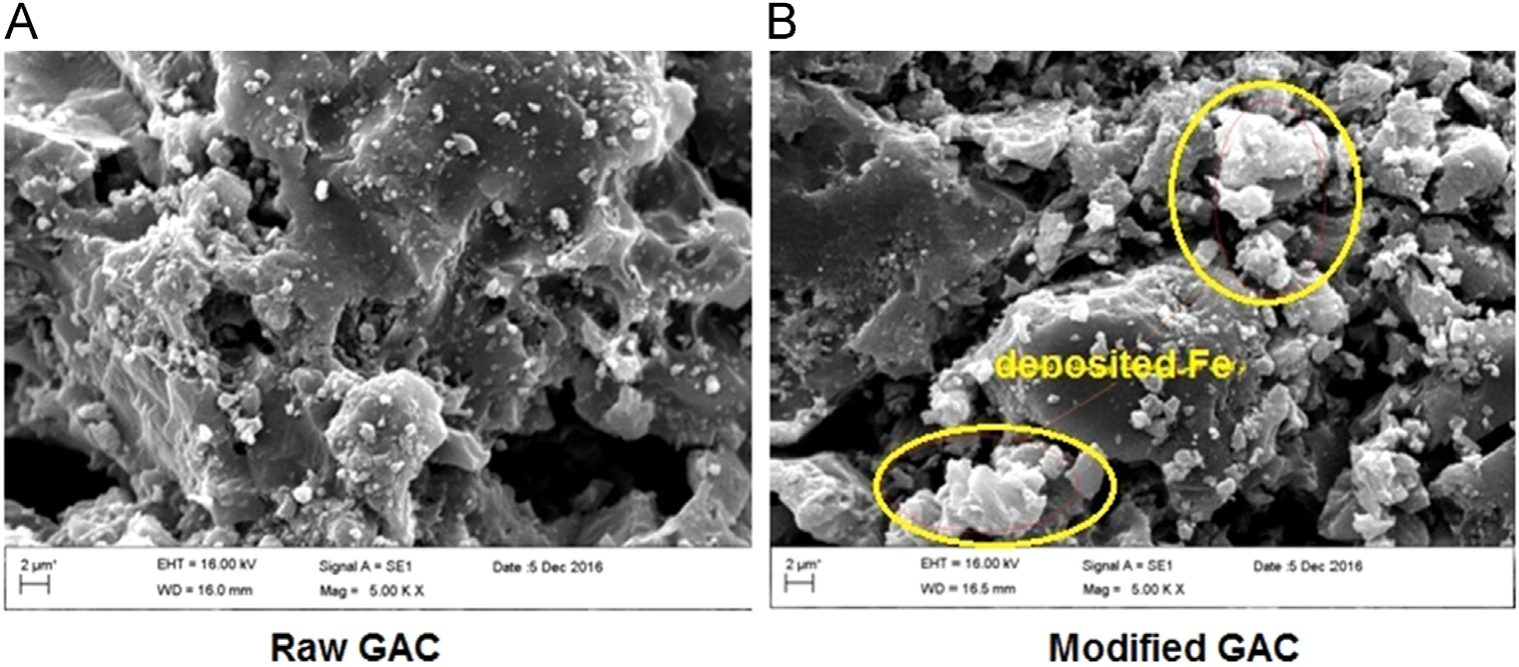
SEM image of raw GAC (A) and modified GAC (B).

**Fig. 2:**
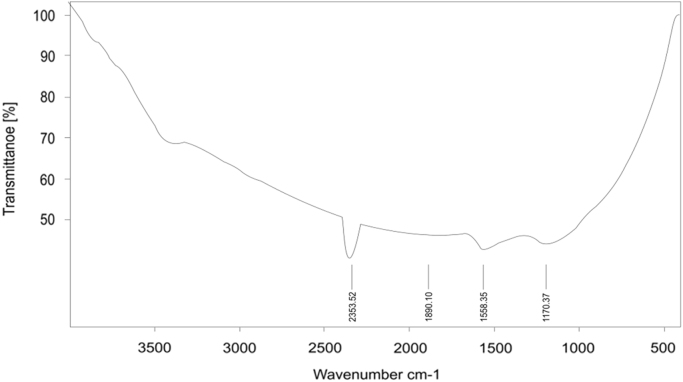
FTIR spectra for raw GAC.

**Fig. 3:**
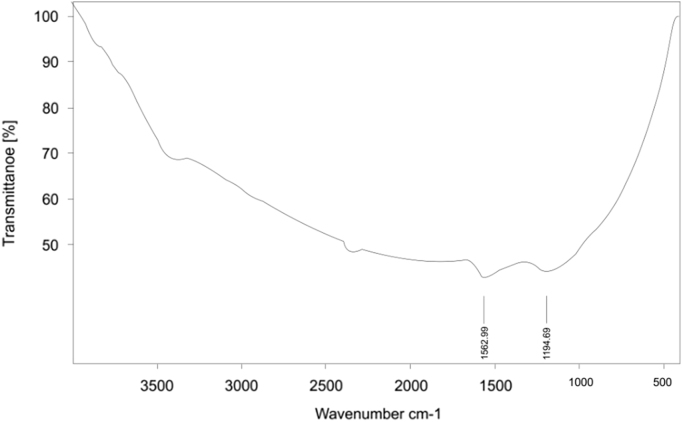
FTIR spectra for modified GAC.

**Fig. 4:**
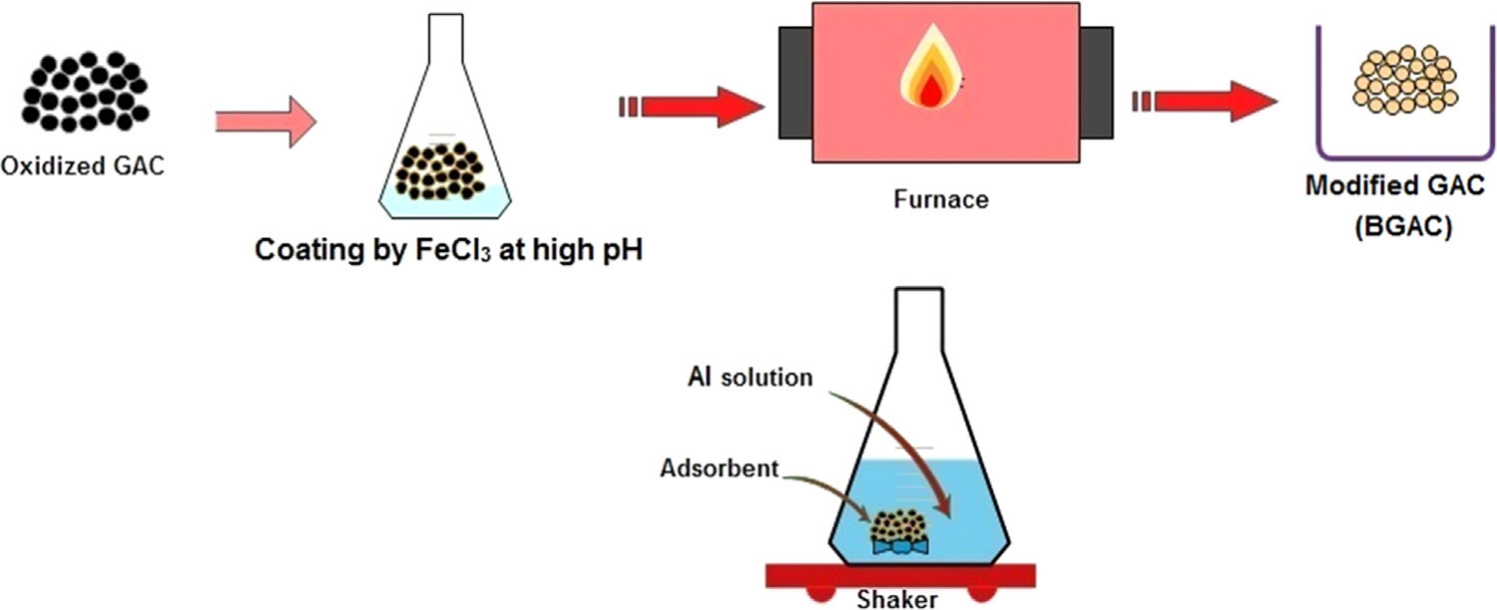
Experimental procedure for GAC modification. GAC Modification was including 1: oxidation by both 65% nitric acid and concentrated sulfuric acid, 2: coating of oxidized GAC by FeCl_3_. 6H_2_O solution containing 2.5% of Fe^3+^ (pH was adjusted to 12 and coating conducted at 80 °C for 24 h), 3: calcination at 300 °C under a N_2_ atmosphere for 3 h, 4: production of modified GAC (BGAC). 5: Batch approach.

**Fig. 5:**
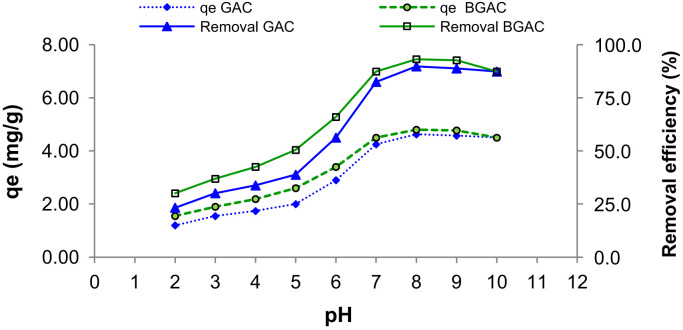
Al removal efficiency and adsorption capacity of raw and modified GAC (BGAC) at different pH. Adsorbents dosage: 2 g/L, Al concentration: 10.3 mg/L, contact time: 24 h and mixing speed: 250 rpm.

**Fig. 6:**
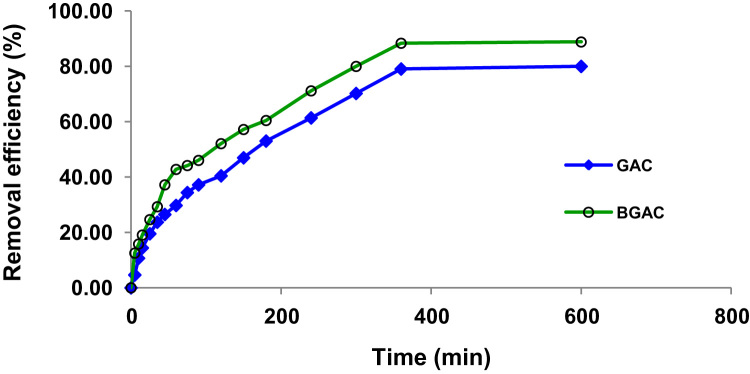
The removal efficiency of Al by raw and modified GAC (BGAC) under different contact time. adsorbents dosage: 2 g/L, Al concentration: 10.3 mg/L, contact time: 24 h and mixing speed: 250 rpm.

**Fig. 7:**
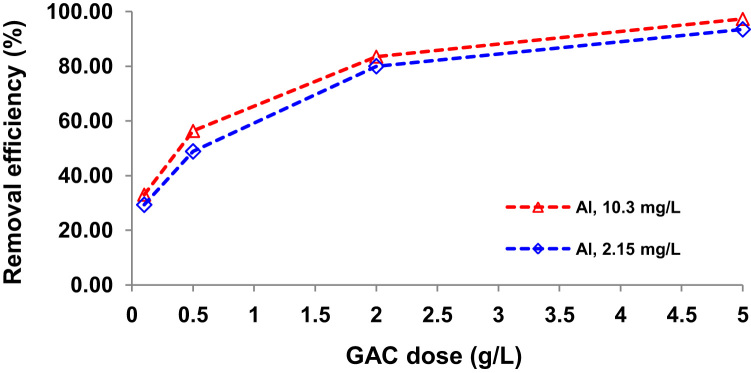
Al removal efficiency by different dosage of raw GAC (0.1, 0.5, 2 and 5 g/L).

**Fig. 8:**
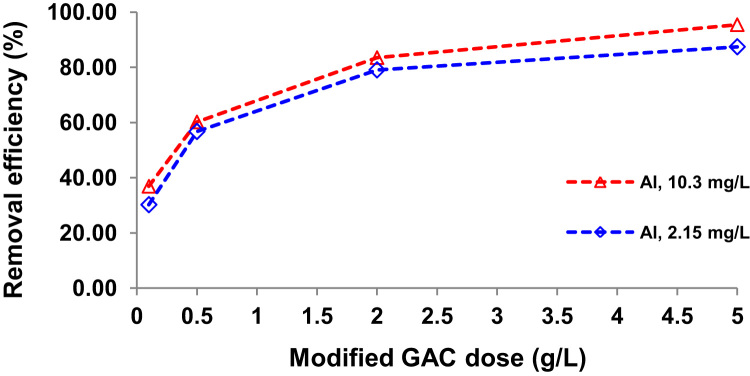
Al removal efficiency by different dosage of modified GAC (0.1, 0.5, 2 and 5 g/L).

**Fig. 9:**
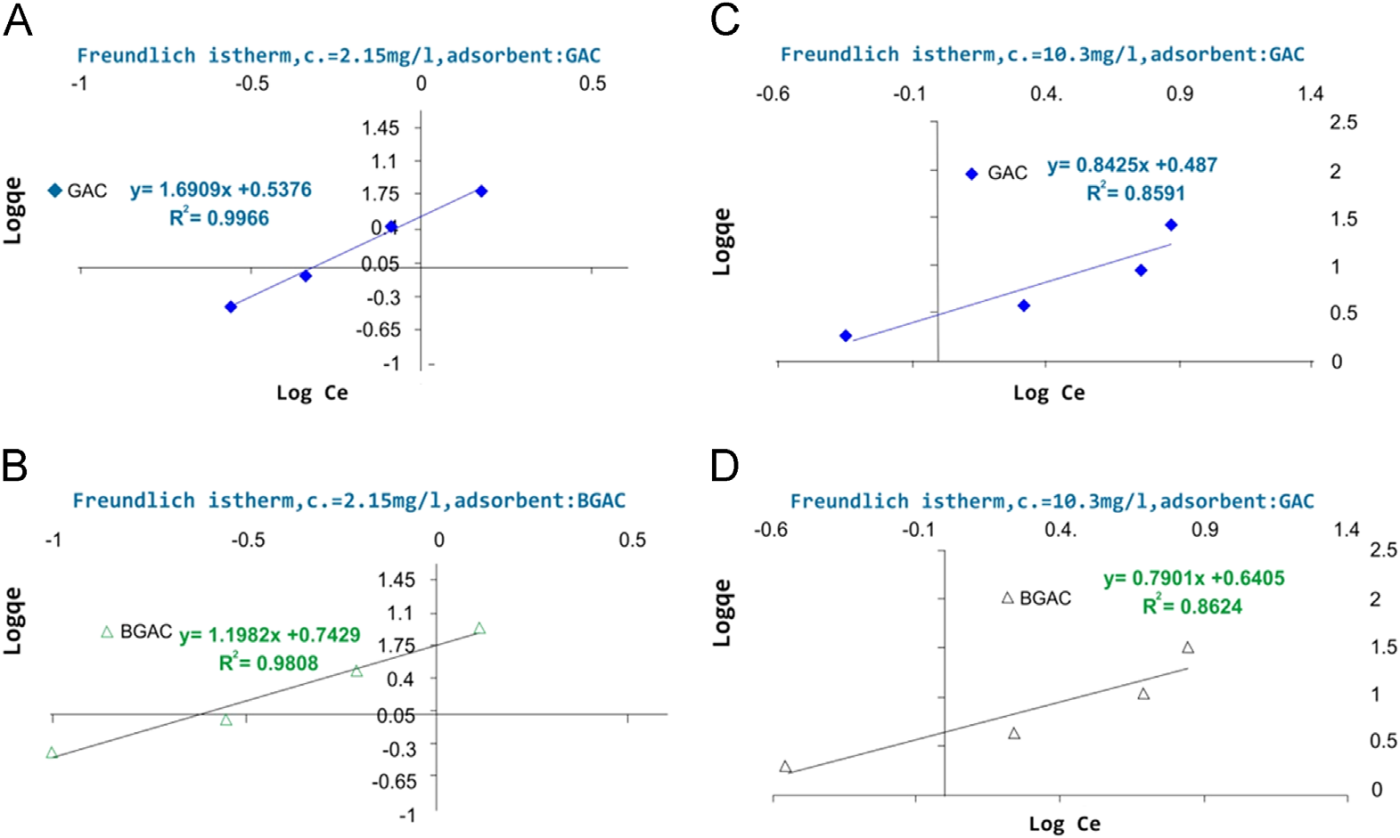
Freundlich isotherm of raw and modified GAC (BGAC). For Part A and B, Al concentration was 2.15 mg/L and adsorbents dose: 0.1, 0.5, 2 and 5 g/L. For Part D and E Al concentration was 10.3 mg/L and adsorbents dose: 0.1, 0.5, 2 and 5 g/L.

**Fig. 10:**
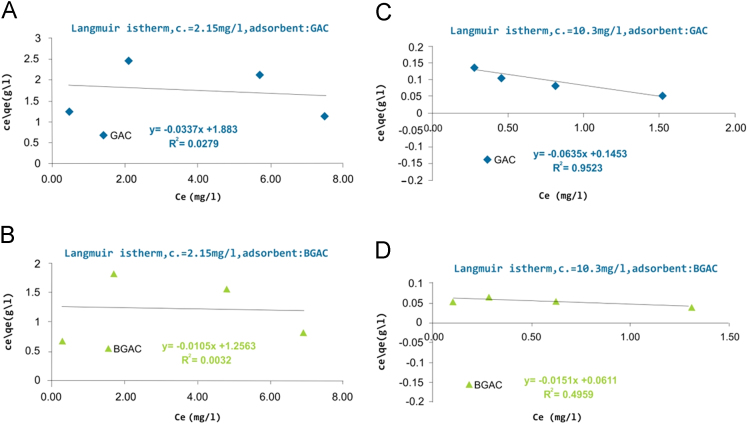
Langmuir isotherm of raw and modified GAC (BGAC). For Part A and B Al concentration was 2.15 mg/L and adsorbents dose: 0.1, 0.5, 2 and 5 g/L. For Part D and E Al concentration was 10.3 mg/L and adsorbents dose: 0.1, 0.5, 2 and 5 g/L.

Presented data in this article comprise the characterization of raw and modified GAC (in this paper modified GAC under basic condition nominated as BGAC) with analytical methods like FTIR, SEM, BET and iron content, as well as experimental data including studying different variables (pH, contact time, Al concentration and adsorbent dosage), isotherm and kinetic. One of the best available technologies for pollutants removal from aqueous solutions is adsorption which has a very good efficiency [1], [2]. Table 1 shows the iron content, BET surface area and other related data about the raw and modified GAC. Fig. 1, Fig. 2, Fig. 3 show the data for SEM and FTIR for raw and modified GAC and Fig. 4 represents the experimental procedures. Kinetics and Isotherms equations presented in Table 2, Table 3 and Kinetics data for Al adsorbed onto raw and modified GAC was presented in Table 4. Fig. 5, Fig. 6, Fig. 7, Fig. 8 show the removal of Al with raw and modified GAC by different parameters. Fig. 9, Fig. 10 shows the adsorption isotherm for Al removal with raw and modified GAC (BGAC).

**Table 1:** Specification of surface area and pore volume of raw and modified GAC with BET test.

**Adsorbent**	**Total volume (cm**^**3**^**/g)**	**BET surface area (m**^**2**^**/g)**	**Total pore volume (cm**^**3**^**/g)**	**Average pore diameter (nm)**	**Fe content**^a^**(mg/g)**
Raw GAC	217/09	944/89	0/4621	1/9564	2.5
Modified GAC (BGAC)	136/43	593/81	0/3114	2/0979	81.2

a $\mathrm{Iron content}=\frac{\mathrm{mass of iron}}{\mathrm{mass of GAC}+\mathrm{mass of iron}}\times 100$.

**Table 2:** Kinetic equations and linear forms used in this work.

**Kinetic**	**Equation**	**Linear form**
Pseudo first order	$\frac{{\mathrm{dq}}_{t}}{\mathrm{dt}}=k_{1}(q_{e}-q_{t})$	${\log\;k}_{1}\left(q_{e}-q_{t}\right)=\log\left(q_{e}\right)-\frac{k_{1}}{2.303}t$
Pseudo second order	$\frac{{\mathrm{dq}}_{t}}{\mathrm{dt}}=k_{2}{(q_{e}-q_{t})}^{2}$	${\frac{t}{q_{t}}}_{1}=\left(\frac{1}{k_{2}q_{e}²}\right)+(\frac{1}{q_{e}})t$
Elovich	$\frac{{\mathrm{dq}}_{t}}{\mathrm{dt}}=\alpha \;\exp(-\beta {q_{t})}^{2}$	$q_{e}=\frac{1}{\beta }\ln(\alpha \beta )+\left(\frac{1}{\beta }\right)\ln t$

**Table 3:** Isotherms equations and linear forms used in this work.

**Type of isotherm**	**Equation**	**Linear form**
Freundlich	$q_{e}=K_{f}\times {\left(C_{e}\right)}^{1/n}$	${\log\;q}_{e}={\log\;K}_{f}+\left(\frac{1}{n}\right)\log\;C_{e}$
Langmuir	$q_{e}=\frac{Q_{m}\times K_{l}C_{e}}{1+K_{l}C_{e}}$	$\frac{C_{e}}{q_{e}}=\left(\frac{1}{K_{l}Q_{m}}\right)+\left(\frac{1}{Q_{m}}\right)C_{e}$

**Table 4:** Kinetics data for Al adsorbed on raw and modified GAC (BGAC). Al concentration was 19.5 mg/L and adsorbents dose was 2 g/L.

Kind of Kinetic	**Parameter**	**GAC**	**BGAC**
Pseudo first order	*q*_e_	9.8	10.66
	*k*_1_	0.009	0.01
	*R*^2^	0.83	0.8
Pseudo second order	*q_e_*	10.07	10.42
	*k*_2_	0.0008	0.0012
	*R*^2^	**0.943**	**0.955**
Elovich	*α*	0.566	0.53
	*β*	0.262	0.39
	*R*^2^	0.916	0.932

## 2 Experimental design, materials and methods

In this work the removal of Al from water was carried out by raw GAC (supplied by the Merck Company) and modified GAC by FeCl_3_ under basic pH condition (BGAC). Some wastewater like spent filter backwash water from water treatment plant was discharged to surface or groundwater without any treatment and it was endangered soil, water body and environment [3], [4], [5], [6], [7], [8]. So it was necessary for all water treatment plants that treat their wastewater before entering to environment.

### 2.1 Materials

Analytical grade ferric chloride (FeCl_3_·6H_2_O), GAC, sulfuric acid, nitric acid and sodium hydroxide were purchased from Merck Company. Also, AlK(SO4)2·12H_2_O was used for aluminium stock solution.

### 2.2 Experiment protocol

#### 2.2.1 Preparation of modified GAC

40 g of the oxidized GAC was mixed with 200 mL of FeCl_3_·6H_2_O solution containing.

2.5% of Fe^3+^ and pH was adjusted to12 by the addition of 1 N NaOH solution. The impregnation of Fe was carried out at 80 °C for 24 h on shaker with 150 rpm rotation [9]. Impregnated GAC was calcined at 300 °C under a N_2_ atmosphere for 3 h. Then it was washed with distilled water for several times and dried at 110 °C during 24 h [10].

#### 2.2.2 Adsorption experiments

Adsorption experiments were conducted by batch method in a 200 mL Erlenmeyer flask and stirred at 250 rpm in a shaker–incubator instrument. Experiments included determination of optimum pH, equilibrium time, dose of adsorbents, Al concentration, the kinetic studies and adsorption isotherms.

For optimum pH selection, 50 mL of Al solution (*C*_o_ = 10.3 mg/L) introduced in 200 mL Erlenmeyer flasks. Then 0.1 g of the adsorbents was put in contact with 50 mL of Al solution (dose of adsorbent was 2 g/L). The pH was adjusted to 2, 3, 4, 6, 7, 8, 9 and 10 by using 1 M HCl or 1 M NaOH (pH was measured by pH-meter model CG 824). The samples were placed in mechanical shaker for 24 h at the room temperature (20 ± 1 °C) and after that, the combination of Al solution and adsorbents was filtered through Whatman paper (0.45 µm) and the concentration of the residual Al was determined by DR-5000.

Percentage removal of Al and adsorption capacity of adsorbent at time *t* (qt) were calculated as Eqs. (1) and (2):

(1) $$\mathrm{Percentage}\;\mathrm{removal}\;\%=\left[1-\frac{C_{e}}{C_{0}}\right]\times 100$$

where *C*_0_ and *C_e_* (mg/L) are the initial and equilibrium solute concentrations, respectively.

(2) $$q_{e}(\mathrm{mg}/g)\;=\left[\frac{C_{0\;}-C_{e}}{M}\right]\times V$$

where *C*_0_ and *C_e_* (mg/L) are the initial and final concentration of Al at time *t* in the solutions, respectively. *M* (g) is the amount of the adsorbent used and *V* (L) the volume of Al solution.

To obtain dataset for adsorption equilibrium isotherms, two initial concentrations of Al (2.15 and 10.3 mg/L) and several doses of adsorbents (0.1, 0.5, 2 and 5 g/L) were used at optimum pH (8) and contact time (6 h).
