# Supplementary material for: Experimental data for aluminum removal from aqueous solution by raw and iron-modified granular activated carbon
Source: Data Brief. 2018 Jan 31;17:731–8. doi: 10.1016/j.dib.2018.01.063 (PMC5988382; doi:10.1016/j.dib.2018.01.063)
Supplement: Supplementary file 1 — Transparency document [file mmc1.doc]

**COVERING LETTER**

| Article Title Experimental data for aluminum removal from aqueous solution by iron-modified granular activated carbon |
| --- |
| **the name of the journal:**  **DATA IN BRIEF** |
| the full names of all authors, clearly indicating the corresponding author   | **Mokhtar Mahdavi ^1,2^** |  | **Afshin Ebrahimi ^3^** | | --- | --- | --- | | **Amir Hossein Mahvi ^4,5^** |  | **Ali Fatehizadeh3** | | **Farham Karakani ^6^** |  | **Hossein Azarpira ^1,2^** |   1- Assistant Professor of Environmental Health Engineering, Saveh University of Medical Sciences, Saveh, Iran. [ShamaLL6@yahoo.com](mailto:ShamaLL6@yahoo.com).  2- Social Determinates of Health Research Center, Saveh University of Medical Sciences, Saveh, Iran.  3- Department of Environmental Health Engineering, Environment Research Center, Research Institute for Primordial Prevention of Non Communicable Disease, Isfahan University of Medical Sciences, Isfahan, Iran. [a_ebrahimi@hlth.mui.ac.ir](mailto:a_ebrahimi@hlth.mui.ac.ir), +983117783247  4- School of Public Health, Tehran University of Medical Science, Tehran, Iran.  5- Center for Solid Waste Research, Institute for Environmental Research, Tehran University of Medical Science, Tehran, Iran. [ahmahvi@yahoo.com](mailto:ahmahvi@yahoo.com)  6- Manager of Passive Defense of Water & Wastewater Engineering Company, Tehran-Iran. [farhamkarakany@yahoo.com](mailto:farhamkarakany@yahoo.com)  * Corresponding author, Hossein Azarpira - Environmental Health Engineering Department, Saveh University of Medical Sciences, Social Determinants of Health Research Center, Saveh, Iran. [hazarpira912@gmail.com](mailto:hazarpira912@gmail.com) +989120572613 |
| **Abstract**  This dataset deals with the modification of granular activated carbon (GAC) with FeCl_3_ under basic conditions (pH ≈12) for removal of aluminium (Al) from aqueous solution. The structural properties and operational parameters including Al ion concentration (2.15 and 10.3 mg/L), pH solution (2-10), adsorbent dosage (0.1- 5 g/L), and contact time (0-10 h) was investigated for raw and modified GAC. This dataset provides information about Al removal by GAC and modified GAC at conditions including: pH = 8, contact time = 6 h, initial Al concentration = 2.15 mg/L. The characterization data of the adsorbents was analysed by Fourier transform infrared (FTIR) spectroscopy, scanning electron microscopy (SEM) and Brunauer, Emmett and Teller (BET) test. The data showed that Freundlich isotherm with and Pseudo second order kinetic model were the best models for describing the Al adsorption reactions. The acquired data indicated that the maximum adsorption capacity of GAC and modified GAC to uptake Al (C_0_=10.3 mg/L) was 3 and 4.37 mg/g respectively. |
| **Key Words**  Aluminim removal, adsorption, iron-modified GAC, water treatment |

| Name and address of corresponding author  Hossein Azarpira - Environmental Health Engineering Department, Saveh University of Medical Sciences, Social Determinants of Health Research Center, Saveh, Iran. [hazarpira912@gmail.com](mailto:hazarpira912@gmail.com) +989120572613 | |
| --- | --- |
| Telephone #  +989120572613 | Fax # |
| Email [hazarpira912@gmail.com](mailto:hazarpira912@gmail.com) | |

I affirm that the manuscript has been prepared in accordance with International Journal of **DATA IN BRIEF** instructions to authors and the content of this manuscript, or a major portion thereof, has not been published in a referred journal no being submitted for publication elsewhere.

Signature of Corresponding Author Date

**12/09/ 2017**

**18/01/ 2018**

**
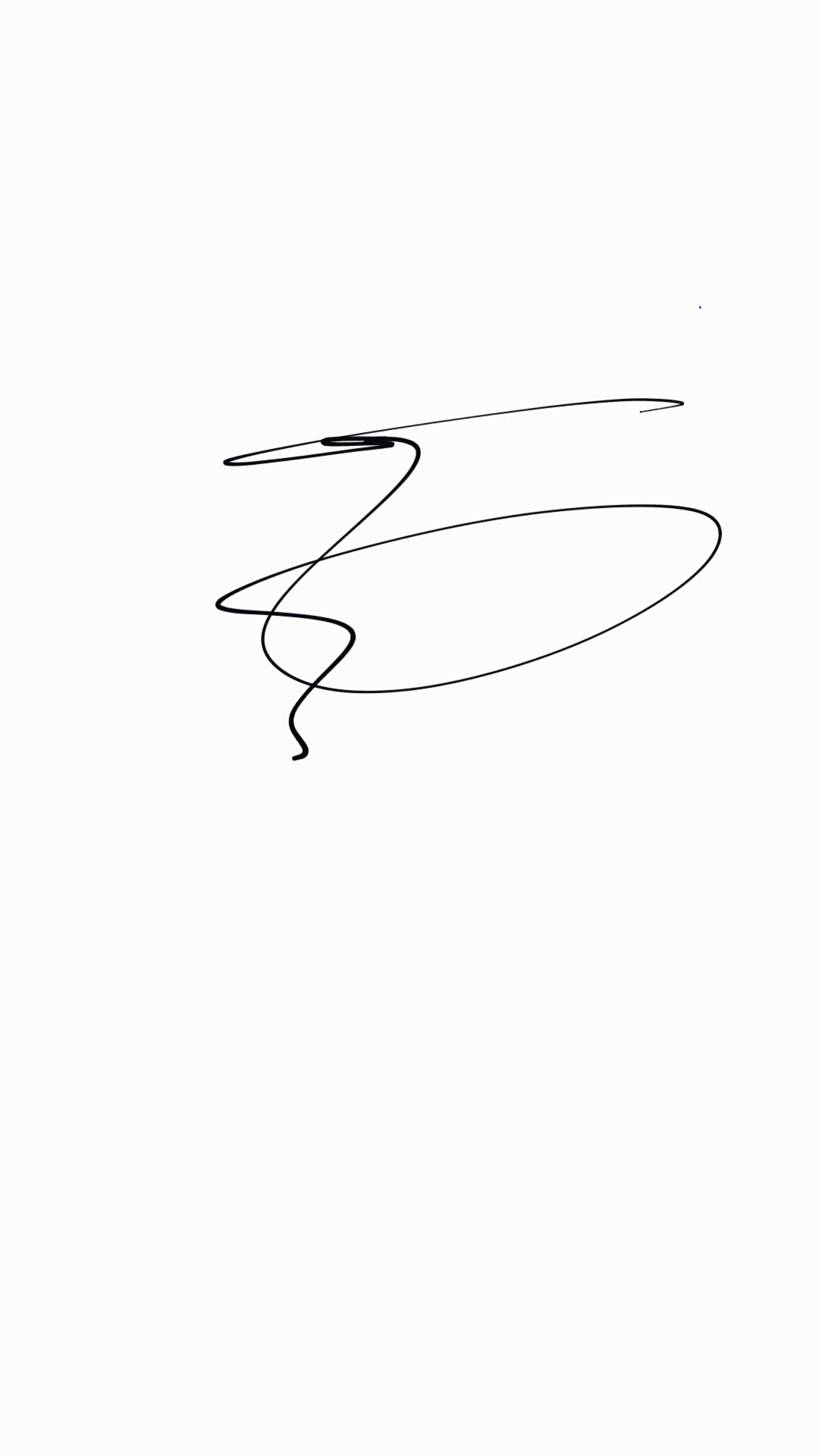
**
